# Extrusion of Irrigant in Open Apex Teeth with Periapical Lesions Following Laser-Activated Irrigation and Passive Ultrasonic Irrigation

**DOI:** 10.22037/iej.v13i2.17150

**Published:** 2018

**Authors:** Harry Huiz Peeters, Ketut Suardita, Latief Mooduto, Norbert Gutknecht

**Affiliations:** a *Laser Research Center in Dentistry, Bandung, Indonesia; *; b * Department of* * Conservative Dentistry, Faculty of Dentistry,* * Universi* *tas* * Airlangga, Surabaya, Indonesia; *; c * Department of* * Conservative Dentistry, Faculty of Dentistry,* * Universi* *tas* * Airlangga, Surabaya, Indonesia; *; d * Department of Operative Dentistry, Periodontology and Pediatric Dentistry, University Hospital RWTH Aachen, Aachen, Germany*

**Keywords:** Endodontics, Er, Cr, YSGG Laser, Open Apex, Periapical Lesion

## Abstract

**Introduction:**

Sodium hypochlorite (NaOCl) irrigation is critical for the success of endodontic treatment and several agitation techniques have been developed to improve the efficacy of this irrigation. Using a combination of contrast medium and radiographic examination, this study evaluated NaOCl extrusion during agitation of irrigant. Development of pressure, which may result in apical extrusion of the irrigant, has been described during laser-activated irrigation (LAI) and passive ultrasonic irrigation (PUI).

**Methods and Materials:**

We examined 40 single root canals categorized as having open apices with apical lesions in 40 patients. For the final irrigation, the teeth were irrigated with a mixture of radiopaque contrast medium and 2.5% NaOCl in solution. The solution was activated for 60 sec in both groups [the Er, Cr: YSGG laser group (*n*=20) and the ultrasonic group (*n*=20)]. The teeth were imaged subsequently using radiography for the evaluation of contrast extrusion.

**Results:**

Radiopaque contrast medium was absent from the periapical tissues in all cases.

**Conclusion:**

Use of LAI or PUI appears to be safe as used currently in endodontic treatment.

## Introduction

An important objective of mechanical instrumentation and irrigation in endodontic therapy is the elimination of microorganisms from the root canal system (RCS) by mechanical and chemical debridement of necrotic tissue. This is especially important in areas of the root canal that have been left unprepared by mechanical instruments. Reinfection must be prevented by complete obturation with an inert filling material. However, after chemomechanical preparation, an amorphous, irregular layer 1 to 2 m thick and known as the smear layer is formed on the RCS, covering the dentinal walls [1-3]. Current methods used to remove the smear layer include chemical, ultrasonic, and laser techniques, none of which is either totally effective or has received universal acceptance [4, 5]. Moreover, in a certain occasion, the debris/smear layer could be accidentally extruded into the apical tissue during instrumentation [6, 7]. 

Several studies have shown that large areas of the canal wall, particularly in the apical third but also in ribbon-shaped and oval canals, cannot be cleaned mechanically, which means that microorganisms that are present in these untouched areas may survive. Residual bacteria and other microorganisms can persist either in these spaces or in tubules. Irrigants and other intracanal medicaments are necessary adjuncts that enhance the antimicrobial effect of mechanical cleansing and thus increase the overall clinical efficacy of the procedure. Irrigation is complementary to instrumentation in facilitating a successful outcome, because it eradicates the bacteria or fungi that are present in the tubules and in the crevices, fins, and ramifications of the RCS [8].

Passive hand irrigation with a needle is the standard cleaning procedure, but unfortunately this is insufficiently effective in the apical third of narrow root canals [9]. Irrigation dynamics play an important role in the effectiveness of irrigation and depend on the mechanism(s) of action of the irrigant and the ability to bring the irrigant into contact with the microorganisms and debris within the root canal space [10]. To enhance the dispersal of the irrigant and to activate it, sonic and ultrasonic techniques have been investigated and developed. Lasers have also been proposed as an alternative to the conventional approach to cleaning and disinfection [11]. Physical techniques to remove the smear layer include the use of ultrasound and pulsed middle infrared lasers, both of which cause cavitation and pressure waves within the root canal space [12]. Various types of laser have been investigated in an attempt to develop improved treatment methods, and the performance of lasers used in the field of dentistry has been improving [13].

Several *in vitro* studies have demonstrated that extrusion of irrigants beyond the apical constriction can occur when root canals are prepared and irrigated [14-17]. These studies provide evidence that irrigating solution can penetrate periapical tissues regardless of the type of irrigant, irrigation delivery device, or activation system used. There are several reports describing the complications of irrigation with NaOCl during root canal therapy [18, 19]. One study showed increased extrusion of debris with higher hypochlorite concentration [20]. Most complications are the result of accidental extrusion of the solution into the periapical area from the apical foramen, accessory canals or perforations [19]. Most NaOCl accidents occur because of inaccurate determination of the working length, iatrogenic widening of the apical foramen, lateral perforation or wedging of the irrigating needle. If a perforation or an open apex is present, particular care is needed during irrigation [21].

The continuity of apical structures surrounding open apices associated with periapical lesions is a controversial topic in endodontics. To the best of our knowledge, few *in vivo* studies have focused on the penetration of irrigating solution into periapical tissues. However, the risk of apical. PUI and LAI methods have recently been proposed to improve irrigation efficacy. Considering the lack of research on these particular conditions, it is important to evaluate teeth with open apices associated with apical lesions. To date, very few studies have investigated extrusion of the irrigating solution from teeth with these particular conditions. Therefore, the aim of this *in vivo* study was to evaluate the safety of different irrigant agitation methods using a combination of contrast medium and radiographic methods.

## Materials and Methods

***Patient selection***

This study was conducted with the approval of the ethical review board for studies on human subjects of Universitas Airlangga, Surabaya, Indonesia (129/KKEPK.FKG.VIII/2012). We declare that all experiments on human subjects were conducted in accordance with the Declaration of Helsinki (http://www.wma.net). The medical history of each patient was obtained from a written health questionnaire, verbal questioning and a clinical examination. Each participant signed an informed consent. Teeth diagnosed with pulpal necrosis in participants of both sexes were selected. The diagnosis was confirmed by a negative response to cold and electric pulp tests; all teeth had radiographically visible chronic periapical lesions. None of the teeth involved in the study demonstrated clinical signs, and periodontal probing revealed no increased probing depth around any of the teeth. Only single-rooted teeth were included. The patients were aged between 21 and 64 years (mean: 36.4 years). Once eligibility had been confirmed, the patients were informed of the study design, clinical procedures involved and associated risks. They were also informed that root canal treatment would be performed regardless of whether they consented to participate in the study. Clinical and radiographic follow-up examinations were undertaken after written informed consent had been provided. Pregnant women, patients who were allergic to any of the components of the formula, those who had teeth with sclerosis, and those who declined to participate in the study were excluded. Finally, the study sample comprised 40 single root canals in incisors, canines and premolars that required routine, nonsurgical endodontic therapy between January 2012 and December 2014. The study was performed by a single experienced endodontist using a standardized protocol.

***Preparation of the radiopaque-NaOCl irrigation solution and irrigation procedure***

A mixture of radiopaque contrast medium and NaOCl in solution was used as the contrast medium to assess the presence or absence of radiopaque materials in the periapical tissues. A sterile intravascular contrast medium (BraccoSpA, Milan, Italy) containing iomeprol (0.81 g/mL) was mixed with 2.5% NaOCl in a ratio of 40:60 by volume to form the final irrigation solution. This mixture had a density and viscosity similar to that of pure NaOCl and permitted the flow of irrigant solution and radiopaque medium to stain the canal wall thoroughly. The irrigant was carefully mixed by a chemist at the Department of Chemistry, Institut Teknologi Bandung, Bandung, Indonesia. For the LAI group, the canals were irrigated by deploying the solution in the pulp chamber, whereas in the PUI group the canals were irrigated 2 mm short of the working length.

**Figure 1:**
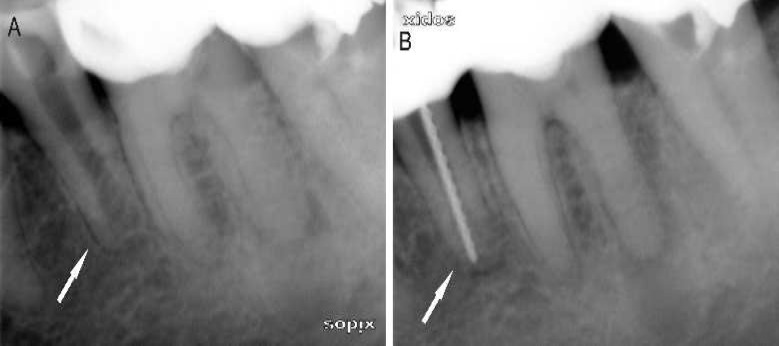
*A*) This canal is categorized as an open canal*; B) *A file size 80 is seen in situ

***Laser parameters and Laser-activation procedure***

Erbium chromium: yttrium-scandium-gallium-garnet (Er,Cr:YSGG) laser was used. The Waterlase MD dental laser (Biolase Technology, San Clemente, CA, USA) had a panel setting of 1 W (average power) at 35 Hz. This laser emits energy in pulses that last approximately 140 μs. The pulses were focused through an MZ2 (plain) tip with a diameter of 200 µm, which was fixed in the laser’s handpiece (corresponding to 28 mJ). The pulse energies were corrected for energy loss using a calibration factor of 0.30 (manufacturer’s information). The actual power output of the system was determined using a laser power meter (FieldMaster GS + Detector LM45, Coherent Inc., Santa Clara, USA) placed at a distance of 10 mm from the terminus of the fiber. According to the laser power meter, the average power at the distal end of the quartz tip was 0.75 W, which yielded a pulse energy of 21.5 mJ/pulse. The fluence (energy density) of the tip was 68.47 J/cm^2^, and the power density of the tip was 1.54 W/cm^2^. The pulp chamber served as a reservoir for the irrigation solution. The tip was submerged in the solution and made to hover above the orifice in the cervical region, rather than inserting the tip into the canal. Coaxial water and air sprays were deactivated. In all cases (*n*=20), the solution was activated for 60 sec.

***PUI procedure***

The patients in the PUI group (*n*=20) underwent the following procedure. A stainless steel non-cutting wire (size=20, Irrisafe, Satelec Acteon, Mérignac, France) was used, driven by an ultrasonic device (Suprasson Pmax Newtron, Satelec Acteon Mérignac, France) at a power setting *blue 5* (frequency, 30 kHz; displacement amplitude, approximately 30 mm; in accordance with the manufacturer’s instructions) for 60 sec. The tip of the ultrasonic device was introduced to 2 mm short of the working length (WL) and activated passively without any filling motion.

**Figure 2:**
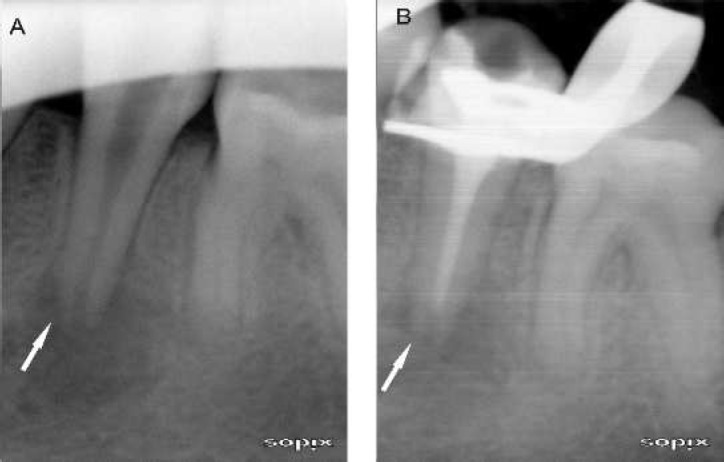
*A*) Before treatment; *B)* The contrast medium has not reached the apex of the root (PUI group)

***Root canal treatment protocol***

The treatments were performed in two visits by an experienced endodontist and using a standardized protocol. In each case, a periapical radiograph was taken to determine the presence of apical lesions, the canal morphology, and the length and number of canals. A routine endodontic therapy procedure was performed on all root canals. Under rubber dam isolation, the pulp chamber was accessed using a diamond bur. Pulp remnants were extirpated and the WL was estimated with the aid of an apex locator (Root ZX®; J Morita USA, Inc., Irvine, CA, USA). No effort was made to enlarge the original apical canal. The final apical preparation was performed using K-files, size 80 (Dentsply Maillefer, Ballaigues, Switzerland), on the basis of the original size of the root canals (Figure 1A). Teeth with an apex smaller than size ISO 80 were excluded from the study, until finally the study comprised 40 root canals with apical lesions. The root canals were cleaned and shaped using K-files with 2.5% NaOCl applied *via* a 28-gauge endodontic needle. At the completion of cleaning and shaping, the smear layer was removed using 3 mL of 17% ethylenediaminetetraacetic acid (EDTA). The final irrigation was accomplished using a mixture of 2.5% NaOCl and contrast medium solution. Subsequently, the irrigant was activated with an Er,Cr: YSGG laser in the laser group and ultrasound in the PUI group. Radiography was performed to observe the presence or absence of contrast medium in the apical tissues. After the canal had been dried with sterile paper points, it was dressed with calcium hydroxide paste (Dycal; Dentsply, De Trey, Konstanz, Germany) using a lentulo spiral instrument. Sterile cotton pellets were placed in the access cavities before they were sealed with a temporary filling (Cavit; 3M ESPE AG, Seefeld, Germany).

In each case, at a second visit 2 weeks later, the teeth remained asymptomatic. The canals were irrigated with 2.5% NaOCl, and a CanalBrush™ (Coltène/Whaledent GmbH, Co. KG, Langenau, Germany) was used to remove the calcium hydroxide. After this procedure, each root canal was dried with sterile paper points and filled with MTA (ProRoot MTA; Dentsply, Tulsa, OK, USA), gutta-percha (Diadent Group International, ChongchongBuk Do, Korea), and AH-Plus sealer (Dentsply, De Trey GmbH, Dentaler zeugnisse, Konstanz, Germany) using the cold lateral compaction technique. Resin-modified glass ionomer cement (GC Fuji II LC; GC Corp, Tokyo, Japan) was placed over the gutta-percha as an orifice plug and the restoration of each tooth was completed with a composite resin (Premisa; Kerr Corp, Orange, CA, USA). 

**Figure 3:**
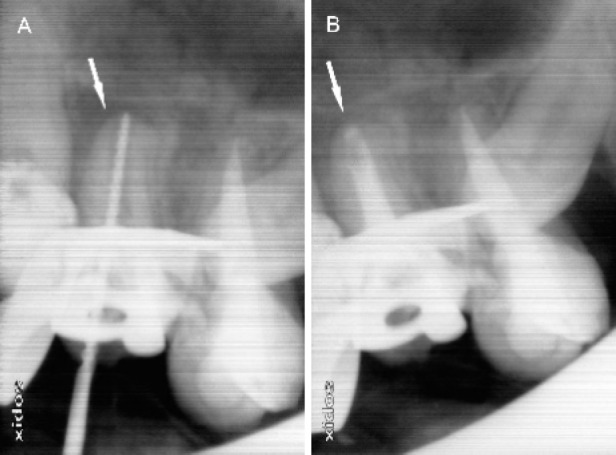
*A* and *B*) Radiographs showing no evidence of radiopaque contrast medium in the periapical tissues (laser group). Note: Arrows indicate the treated teeth

Postoperative radiography was used to check the quality of the filling and to observe the presence or absence of the contrast medium in apical tissues (Figure 1B).

***Radiographic technique***

The teeth were evaluated clinically and radiographically using digital radiography (periapical radiography). X-rays were generated by a Nomad Pro Handheld X-ray System (Arbitex LLC, Bountiful, UT, USA) operating at 60 kVp and 2.5 mA. The exposure time used was 0.13 sec, the minimal focal spot was 0.4 mm and the x-ray field size was 20 cm. Radiographic images of the teeth were obtained using a size #2 intra-oral Sopix CCD image receptor (Sopro; Acteon, La Ciotat Cedex, France). Digital images were saved in TIFF format for future evaluation. Images obtained following use of the irrigant containing radiopaque solution were investigated for the presence of apical extrusion of irrigant into the periapical tissues. 

***Assessment of the treatment***

The root canals with final apical preparation using file sizes ISO 50-80 were categorized as large apices, and those using size ISO>80 were categorized as open apices. The detailed categorization of the final apical preparation was reported previously [24]. The study involved 40 patients who required routine, nonsurgical endodontic therapy between January 2012 and December 2014. The study sample comprised 40 open root canals in incisors, canines and premolars with periapical lesions. At the final examination (second appointment), pain, depth of the pocket, swelling, findings on gingival palpation, and sinus tracts were recorded. The outcome was recorded as either ‘no’ (absence) if the radiopaque medium was not present in the periapical tissues, or ‘yes’ (presence) if radiopaque medium was present in the periapical tissues. Three calibrated and blinded radiologists independently determined the presence or absence of radiopaque medium in the periapical tissues. Where there were differences between their assessments, the three examiners reached a consensus.

**Figure 4:**
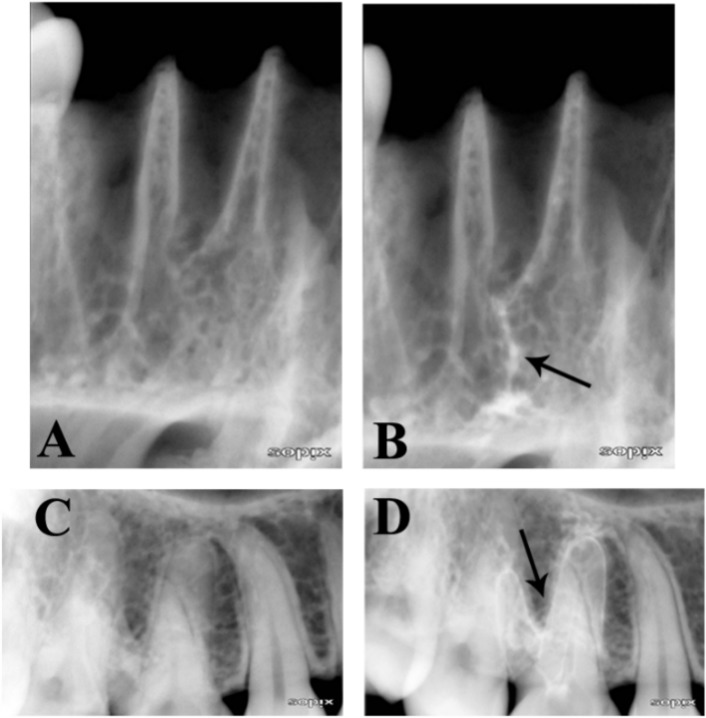
Radiographs showing the evidence of radiopaque contrast medium in the periapical tissues (cadaver). Note: Arrows indicate the contrast medium before (*A* and *C*) and after (*B* and *D*)

***Statistical analysis***

The presence or absence (yes/no) of apical extrusion of contrast medium into the periapical tissues was investigated in all cases. The results were analyzed using the *Chi* squared test. The results were expressed as the number and the percentages of cases which the contrast medium reaching the apex in each group. The Cohen kappa analysis was used to analyze agreement between the three examiners. Statistical analysis was performed using SPSS/PC Statistics Software (SPSS version 18.0, SPSS, Chicago, IL, USA). 

The significance level was set at 0.05.

## Results

After a thorough examination, all teeth were found to be free of pain, swelling, sinus tracts and tenderness to percussion, and the periodontal probing depth was normal when compared with baseline measurements. The value of kappa obtained was 1.00. The Cohen kappa statistic showed excellent intra- and inter-examiner agreement. This suggests that the reliability and reproducibility of the examiners was excellent. On radiographic examination using the radiopaque contrast medium, in the laser group the contrast had reached the apex successfully in 20 teeth. In the PUI group, the contrast had reached the apex successfully in only 9 teeth; in 11 teeth it had not reached the apex (Table 1). No extrusion of irrigant had occurred beyond the apical constriction in teeth with any condition of the roots or periapical tissues during cavitation induced by the Er,Cr: YSGG laser or PUI (Figures 2 and 3).

## Discussion

Various methods have been used to evaluate apical extrusion of irrigants either *in vitro* or *in vivo*; however, this *in vivo* study used a combination of contrast medium and radiography. Iomeprol solution was used as the radiographic contrast medium because it belongs to a new generation of nonionic contrast agents that are water soluble, nontoxic, pyrogen-free, and marketed in a sterile solution. It is widely used in disciplines such as neuroradiology, angiography and urography, and as an enhancing agent in computerized axial tomography; however, it is rarely used in dentistry. In this investigation, it was combined with 2.5% NaOCl, creating a contrast medium that enabled radiographic assessment of the presence or absence of contrast medium in the periapical tissues. A low concentration of NaOCl solution was used in this study because there are reportedly no differences in the optical properties of a low concentration of sodium hypochlorite solution and EDTA solution [25]. In a pilot study, conducted by authors, using a cadaver, we found that the presence of the mixture of radiopaque contrast medium and NaOCl in solution used in the study was obviously seen in radiographic examinations when it was deliberately injected into the tissue (Figure 4).

**Table 1:** Number and percentages of cases which the contrast medium reaching the apex

**Group (N)**	**Reach the apex**		***P*** **-value**
	**Yes**	**No**	
LAI (20)	20 (100%)	0 (0%)	<0.05*
PUI (20)	9 (45%)	11 (55%)	

*LAI: Laser-activated irrigation; PUI: Passive ultrasonic irrigation*

* *Chi-square test; the significance value was set up at 0.05*

A recent study involving evaluation by scanning electron microscopy revealed that an LAI system (1 W, 35 Hz) had the ability to remove the smear layer at the tip of the root [26]. This capacity was attributed to the ability of the LAI system to cause cavitation and fluid streaming. LAI overcame the airlock effect by releasing air trapped in the air column. The mechanism 

underlying the removal of trapped air from the apical region using an Er,Cr: YSGG laser in a dry root canal is *via* the disruption of the surface tension at the solution-air interface. This disruption, caused by bubble implosion (cavitation), displaces air in the form of bubbles from the apical region towards the solution, which allows the solution to travel apically [27]. In addition, bubbles formed during cavitation expand, become unstable and then collapse in a process called implosion [11]. This implosion has an impact on the surfaces of the root canal, causing shear forces, surface deformation and the removal of surface material [28]. Cavitation is defined as the formation of vapor or a cavity containing bubbles inside a fluid [29].

In the present study, we used teeth with periapical lesions surrounding open apices because extrusion accidents are more likely in teeth with non-vital pulp and periapical lesions [30]. This type of apex (lack of resistance to apical flow) or the presence of apical anomaly should have increased the likelihood of apical extrusion of irrigant beyond the apical foramen. However, our results showed no evidence of apical extrusion in the two groups after the procedure had been completed (Figures 1A and 1B), even though the images of large lesions revealed the presence of radiolucent spaces in the periapical tissues. This result is in accordance with a clinical study reported by Peeters and Mooduto [24], who demonstrated that the irrigation solution could be driven by laser activation to the apex without harming the periapical tissues. 

In the laser-treated group, the contrast medium reached the whole of the apical region in 20 cases. This may be because the use of LAI results in a fluid stream toward the apex [11]. Using cinematic holography, Ebeling and Lauterborn [31] observed shock waves that emanated from collapsing bubbles generated by a laser pulse. These laser-generated pressure waves caused fluid movement at high speed and appeared to enhance the action of endodontic irrigants in the removal of the smear layer [22]. When pressure waves cause transverse movement of fluid, the possibility of accidental extrusion of the irrigant beyond the apical constriction is greater with a laser than with an ultrasonic device. In order to avoid some of the side effects associated with the use of a laser, the LAI was performed by hovering the laser tip over the orifice of the root canal system instead of placing the tip within the canal itself. Furthermore, a recent study conducted by Peeters and De Moor [32] concluded that the apical pressure attained during LAI [5.8 (1.0) mm Hg at 1 W] was comparable with the lymphatic capillary pressure when the tip was placed at the cervical region, and could therefore be considered safe.

In radiographic examination, using radiopaque contrast medium, of the PUI group, the contrast did not reach the apex in 11 cases. This is not surprising, because the vibrating file of the ultrasonic devices used in PUI only oscillates in a transverse direction and not longitudinally towards the root axis. Nevertheless, the apical movement of the solution may be attributed to ultrasonically induced wave generation at the solution-air interface, which results in the removal of trapped air from the root canal and allows the solution to travel apically, in the opposite direction [33].

In addition, the authors speculate that the absence of contrast medium in the periapical tissues may also indicate that these tissues remained intact, although despite this there is no indication that the normal apical periodontium still existed in this case. A radiolucent space in the apical tissues indicates that the integrity of hard tissues such as bone, cementum, and dentin has been disturbed. This characteristic radiolucent feature is caused by apical bone resorption. However, histopathologic studies of randomly selected samples have revealed that the apical lesion consists of granulomatous tissue; a fibrous capsule or a plug-like seal may form at some apical foramina as a result of invagination of the epithelium into the apex [8]. Although the results of the current study showed no apical extrusion of irrigant into periapical tissues during LAI and PUI, clinicians should adhere to the highest standards for clinical procedures to avoid iatrogenic accidents. 

Our findings contradict those of Salzgeber and Brilliant [34], who demonstrated using radiographic examination that irrigating solutions reach the periapical tissues sooner during the cleaning and shaping procedure in necrotic teeth compared to the vital teeth. We assume that Salzgeber and Brilliant [34] conducted root canal enlargement while the irrigating solution mixed with contrast agent was in the canal. Furthermore, several other studies have demonstrated that all instrumentation techniques produce extrusion of debris through the periapical region [15, 35-37]. In other words, the solution penetrates the periapical tissues as a result of the unintentional extrusion of debris and irrigant beyond the apex constriction. It is probable that, during the irrigation procedure, the solution does not move easily in an apical direction because of the airlock effect in the closed system [38]. In contrast to the aforementioned study [34], we conducted root canal enlargement separately from the PUI or LAI procedure.

## Conclusion

Radiographic evaluation of the extrusion of radiopaque irrigation solution during PUI and LAI with the fiber tip hovering over the orifice confirmed that no contrast medium was detected in periapical lesions. In other words, use of PUI and LAI to enhance irrigation during root canal treatment under the conditions of the present *in vivo* study appears to be safe. Although this preliminary study did not detect contrast agent in the periapical tissues, it is reasonable to recommend that, during root canal enlargement, instruments should not disrupt the resistance of intact periapical tissues.

## References

[1] Moon YM, Shon WJ, Baek SH, Bae KS, Kum KY, Lee WC (2010). Effect of Final Irrigation Regimen on Sealer Penetration in Curved Root Canals. J Endod.

[2] Liu JN, Kuah HG, Chen NN (2007). Effect of EDTA with and without Surfactants or Ultrasonics on Removal of Smear Layer. J Endod.

[3] Pashley DH (1992). Smear layer: overview of structure and function.

[4] Torabinejad M, Handysides R, Khademi AA, Bakland LK (2002). Clinical implications of the smear layer in endodontics: A review. Oral Surg Oral Med Oral Pathol Oral Radiol Endod.

[5] Torabinejad M, Khademi AA, Babagoli J, Cho Y, Johnson WB, Bozhilov K, Kim J, Shabahang S (2003). A New Solution for the Removal of the Smear Layer. J Endod.

[6] Karade P, Chopade R, Patil S, Hoshing U, Rao M, Rane N, Chopade A, Kulkarni A (2017). Efficiency of Different Endodontic Irrigation and Activation Systems in Removal of the Smear Layer: A Scanning Electron Microscopy Study. Iran Endod J.

[7] Labbaf H, Moghadam KN, Shahab S, Bassir MM, Fahimi MA (2017). An In vitro Comparison of Apically Extruded Debris Using Reciproc, ProTaper Universal, Neolix and Hyflex in Curved Canals. Iran Endod J.

[8] Cohen S, Hargreaves KM (2006). Pathways of the pulp.

[9] Druttman ACS, Stock CJR (1989). An in vitro comparison of ultrasonic and conventional methods of irrigant replacement. Int Endod J.

[10] Gao Y, Haapasalo M, Shen Y, Wu H, Li B, Ruse ND, Zhou X (2009). Development and Validation of a Three-dimensional Computational Fluid Dynamics Model of Root Canal Irrigation. J Endod.

[11] Blanken J, Moor RJGD, Meire M, Verdaasdonk R (2009). Laser induced explosive vapor and cavitation resulting in effective irrigation of the root canal Part 1: A visualization study. Lasers Surg Med.

[12] George R, Rutley EB, Walsh LJ (2008). Evaluation of Smear Layer: A Comparison of Automated Image Analysis versus Expert Observers. J Endod.

[13] Yamazaki R, Goya C, Yu D-G, Kimura Y, Matsumoto K (2001). Effects of Erbium, Chromium:YSGG Laser Irradiation on Root Canal Walls: A Scanning Electron Microscopic and Thermographic Study. J Endod.

[14] Lambrianidis T, Tosounidou E, Tzoanopoulou M (2001). The Effect of Maintaining Apical Patency on Periapical Extrusion. J Endod.

[15] Tinaz AC, Alacam T, Uzun O, Maden M, Kayaoglu G (2005). The Effect of Disruption of Apical Constriction on Periapical Extrusion. J Endod.

[16] Brown DC, Moore BK, Brown CE, Newton CW (1995). An in vitro study of apical extrusion of sodium hypochlorite during endodontic canal preparation. J Endod.

[17] Myers GL, Montgomery S (1991). A comparison of weights of debris extruded apically by conventional filing and canal master techniques. J Endod.

[18] Pashley EL, Birdsong NL, Bowman K, Pashley DH (1985). Cytotoxic effects of NaOCl on vital tissue. J Endod.

[19] Hülsmann M, Rödig T, Nordmeyer S (2007). Complications during root canal irrigation. Endod Topics.

[20] Parirokh M, Jalali S, Haghdoost AA, Abbott PV (2012). Comparison of the Effect of Various Irrigants on Apically Extruded Debris after Root Canal Preparation. J Endod.

[21] Mehdipour O, Kleier DJ, Averbach RE (2007). Anatomy of sodium hypochlorite accidents. Compend Contin Educ Dent.

[22] George R, Meyers IA, Walsh LJ (2008). Laser Activation of Endodontic Irrigants with Improved Conical Laser Fiber Tips for Removing Smear Layer in the Apical Third of the Root Canal. J Endod.

[23] Rodríguez-Figueroa C, McClanahan SB, Bowles WR (2014). Spectrophotometric Determination of Irrigant Extrusion Using Passive Ultrasonic Irrigation, EndoActivator, or Syringe Irrigation. J Endod.

[24] Peeters HH, Mooduto L (2013). Radiographic examination of apical extrusion of root canal irrigants during cavitation induced by Er,Cr:YSGG laser irradiation: an in vivo study. Clin Oral Investig.

[25] Meire MA, Poelman D, Moor RJD (2014). Optical properties of root canal irrigants in the 300–3,000-nm wavelength region. Lasers Med Sci.

[26] Peeters HH, Suardita K (2011). Efficacy of Smear Layer Removal at the Root Tip by Using Ethylenediaminetetraacetic Acid and Erbium, Chromium: Yttrium, Scandium, Gallium Garnet Laser. J Endod.

[27] Peeters HH, Moor RJGD, Suharto D (2015). Visualization of removal of trapped air from the apical region in simulated root canals by laser-activated irrigation using an Er,Cr:YSGG laser. Lasers Med Sci.

[28] Tomita Y, Shima A (1986). Mechanisms of impulsive pressure generation and damage pit formation by bubble collapse. J Fluid Mech.

[29] Hmud R, Kahler WA, George R, Walsh LJ (2010). Cavitational Effects in Aqueous Endodontic Irrigants Generated by Near-infrared Lasers. J Endod.

[30] Kleier DJ, Averbach RE, Mehdipour O (2008). The Sodium Hypochlorite Accident: Experience of Diplomates of the American Board of Endodontics. J Endod.

[31] Ebeling KJ, Lauterborn W (1977). High speed holocinematography using spatial multiplexing for image separation. Opt Commun.

[32] Peeters HH, Moor RJGD (2015). Measurement of pressure changes during laser-activated irrigant by an erbium, chromium: yttrium, scandium, gallium, garnet laser. Lasers Med Sci.

[33] Peeters HH, Iskandar B, Suardita K, Suharto D (2014). Visualization of Removal of Trapped Air from the Apical Region of the Straight Root Canal Models Generating 2-phase Intermittent Counter Flow during Ultrasonically Activated Irrigation. J Endod.

[34] Salzgeber RM, Brilliant JD (1977). An in vivo evaluation of the penetration of an irrigating solution in root canals. J Endod.

[35] Koçak S, Koçak MM, Sağlam BC, Türker SA, Sağsen B, Er Ö (2013). Apical Extrusion of Debris Using Self-Adjusting File, Reciprocating Single-file, and 2 Rotary Instrumentation Systems. J Endod.

[36] Ferraz CCR, Gomes NV, Gomes BPFA, Zaia AA, Teixeira FB, Souza-Filho FJ (2001). Apical extrusion of debris and irrigants using two hand and three engine-driven instrumentation techniques. Int Endod J.

[37] Kuştarcı A, Akpınar KE, Sümer Z, Er K, Bek B (2008). Apical extrusion of intracanal bacteria following use of various instrumentation techniques. Int Endod J.

[38] Peeters HH, Gutknecht N (2014). Efficacy of laser-driven irrigation versus ultrasonic in removing an airlock from the apical third of a narrow root canal. Aust Endod J.

